# Recovery of limb perfusion and function after hindlimb ischemia is impaired by arterial calcification

**DOI:** 10.14814/phy2.15008

**Published:** 2021-08-17

**Authors:** Sara L. Zettervall, Xue‐Lin Wang, Stephanie Monk, Tonghui Lin, Yujun Cai, Raul J. Guzman

**Affiliations:** ^1^ Division of Vascular and Endovascular Surgery Department of Surgery Beth Israel Deaconess Medical Center Harvard Medical School Boston Massachusetts USA; ^2^ Division of Vascular Surgery and Endovascular Therapy Department of Surgery Yale University School of Medicine New Haven Connecticut USA

**Keywords:** arterial calcification, hindlimb ischemia, vascular smooth muscle cells

## Abstract

Medial artery calcification results from deposition of calcium hydroxyapatite crystals on elastin layers, and osteogenic changes in vascular smooth muscle cells. It is highly prevalent in patients with chronic kidney disease, diabetes, and peripheral artery disease (PAD), and when identified in lower extremity vessels, it is associated with increased amputation rates. This study aims to evaluate the effects of medial calcification on perfusion and functional recovery after hindlimb ischemia in rats. Medial artery calcification and acute limb ischemia were induced by vitamin D_3_ (VitD_3_) injection and femoral artery ligation in rats. VitD_3_ injection robustly induced calcification in the medial layer of femoral arteries in vivo. Laser Doppler perfusion imaging revealed that perfusion decreased and then partially recovered after hindlimb ischemia in vehicle‐injected rats. In contrast, VitD_3_‐injected rats showed markedly impaired recovery of perfusion following limb ischemia. Accordingly, rats with medial calcification showed worse ischemia scores and delayed functional recovery compared with controls. Immunohistochemical and histological staining did not show differences in capillary density or muscle morphology between VitD_3_‐ and vehicle‐injected rats at 28 days after femoral artery ligation. The evaluation of cardiac and hemodynamic parameters showed that arterial stiffness was increased while cardiac function was preserved in VitD_3_‐injected rats. These findings suggest that medial calcification may contribute to impaired perfusion in PAD by altering vascular compliance, however, the specific mechanisms remain poorly understood. Reducing or slowing the progression of arterial calcification in patients with PAD may improve clinical outcomes.

## Graphical abstract

Medial calcification is associated with poor outcomes in patients with peripheral artery disease. Rats with medial calcification showed delayed recovery of perfusion and function after femoral artery ligation. These findings were associated with increased arterial stiffness, but no changes in cardiac function or muscle histology.

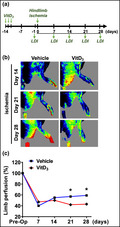

## 1 INTRODUCTION

When arterial calcification is identified in the medial layer, it is associated with decreased survival and increased amputation rates (Ho & Shanahan, 2016). Medial artery calcification is highly prevalent in patients with chronic kidney disease, diabetes, and peripheral artery disease (PAD) (Blacher et al., 2001; Burke, 2004; Eddington et al., 2009; Guzman et al., 2013). It has been shown that calcification in lower extremity arteries is associated with increased adverse outcomes (Huang et al., 2014; Lehto et al., 1996a). We have found that these associations are significant, and remain so even after adjusting for cardiovascular risk factors and the ankle–brachial index in patients with PAD (Guzman et al., 2008).

Through intensive efforts by numerous investigators, the mechanisms that control medial artery calcification have begun to come into focus. It is now known to be a complex process that advances through an imbalance between endogenous stimulators and inhibitors. This results in differentiation of vascular smooth muscle cells into a more osteochondrogenic cell type, along with increased deposition of calcium hydroxyapatite crystals onto the medial layers (Demer & Tintut, 2014; Giachelli, 2009). Associated with these changes in the vascular matrix is a progressive stiffening of the arterial wall related to alterations in medial elastin. Such changes in compliance are thought to lead to decreased end‐organ perfusion (Fortier & Agharazii, 2016). Despite our increased understanding of the molecular processes and structural vessel alterations related to medial calcification, the mechanisms that connect them to worsened ischemic outcomes have not been established.

The study of medial calcification has been aided by the development of rodent models. These experimental systems rely primarily on creating a serum biochemical profile similar to that observed in patients with calciphylaxis. In this model, the fat‐soluble secosteroid cholecalciferol, also known as vitamin D_3_ (VitD_3_), induces calcification in the arterial media when injected at high doses (Bas et al., 2006; Hsu et al., 2008; Mizobuchi et al., 2007). The calcification observed in this model is similar to the medial artery calcification seen in patients with diabetes and chronic kidney disease (Bas et al., 2006; Price et al., 2000). Ligation of the femoral artery with or without partial vessel excision is commonly used to induce limb ischemia in rodents (Brenes et al., 2012; Niiyama et al., 2009). In this model, blood flow and limb function are markedly decreased, and this is followed by gradual recovery to a level that depends on the extent of vessel excised.

The effect of medial calcification on perfusion and functional recovery in ischemic limbs has not been fully explored. We thus used a model that combined VitD_3_ injection and femoral artery ligation to assess the effects of calcification on limb perfusion and functional recovery in rats. In the present study, we demonstrate that animals with medial calcification induced by VitD_3_ show impaired recovery from ischemia. We further provide evidence that increased arterial stiffness as seen with medial calcification may play a role in this process. This study provides impetus for inhibiting arterial calcification to improve outcomes in patients with PAD.

## 2 METHODS

### 2.1 Animals

All animal care and related procedures were performed in accordance with protocols that were approved by the University Animal Care and Use Committee of Beth Israel Deaconess Medical Center and the Joslin Diabetes Centers. Twelve‐week‐old male Sprague Dawley rats (Charles River) were used for all experimental studies. Animals were housed in temperature‐controlled rooms and fed with a normal chow.

### 2.2 Animal models

VitD_3_ injection was used to induce medial calcification as described previously (Marshall et al., 2019; Qin et al., 2006). Briefly, rats were subcutaneously injected with 3 × 10^5^ IU/kg VitD_3_ for 3 consecutive days. VitD_3_ was dissolved in denatured alcohol, Kolliphor EL, and dextrose solution, successively. Control animals were injected with denatured alcohol/Kolliphor EL/dextrose without VitD_3_. In addition to VitD_3_ treatment, a rat model of hindlimb ischemia was used (Luo et al., 2002). Briefly, animals underwent left femoral artery ligation and excision on day 14 after VitD_3_ or vehicle injection. The left common femoral artery was exposed, isolated, doubly ligated, and the vessel segment between the ligatures was excised. The incision was then closed and animals were allowed to recover with normal access to food and water. In a separate experiment, animals were injected with three doses of VitD_3_ and the femoral artery was excised after 14 days for histological analysis.

### 2.3 Laser Doppler perfusion Imaging

Blood flow was measured by laser Doppler imaging in both legs preoperatively and postoperatively at days 7–28 after hindlimb ischemia surgery using a PeriScan PIM II laser Doppler Perfusion Imager (Perimed AB). Animals were anesthetized with 2.5% isoflurane for all perfusion imaging procedures. All animals were kept on a 37℃ heating pad to minimize temperature change and hindlimb hair was removed by hair clipper. Each limb was compared at the same time of day in uniform lighting throughout the study period. Mean hindlimb perfusion was measured for the dorsal foot at a standardized distance by computer‐generated image analysis. Raw perfusion was evaluated for each limb.

### 2.4 Limb functional scoring

The Tarlov score, Ischemia score, and Modified Ischemia Score (Table 1) were utilized to evaluate functional recovery as previously described (Brenes et al., 2012; Tarlov, 1954; Westvik et al., 2009) The total score was derived from the sum of the three scoring modalities. Each functional score was examined prior to ligation, and at days 7, 14, 21, and 28 after hindlimb ischemia. The measurements were performed by a clinical investigator blinded to animal group assignments.

**TABLE 1:** Functional scoring rubric

Score	Tarlov	Ischemia	Modified ischemia
0	No movement	Auto‐amputation: greater than half of lower limb	Auto‐amputation of leg
1	Non‐weight bearing, minimal movement	Gangrenous tissues: greater than half foot	Leg necrosis
2	Non‐weight bearing, frequent movement	Gangrenous tissue: less than half foot, with muscle necrosis	Foot necrosis
3	Partial weight bearing, supports weight	Gangrenous tissue: less than half foot, no muscle necrosis	Discoloration of more than two toes
4	Walks with mild deficit	Pale foot or gait abnormality	Discoloration of one toe
5	Normal slow walking	Normal	Discoloration of more than two nails
6	Normal fast walking	–	Discoloration of one nail
7	–	–	No necrosis

### 2.5 Calcification assessment

Calcium content was measured in arteries using the o‐cresolphthalein complexone method as previously described (Qin et al., 2006). After 14 days of VitD_3_ or vehicle injection, the femoral arteries were isolated, dried, and decalcified in 0.6 N hydrogen chloride for 24 h. Calcium content was expressed as µg/mg dry tissue.

### 2.6 Quantitative PCR

Quantitative PCR (qPCR) was used to determine mRNA levels of osteogenic and smooth muscle markers. Total RNA was isolated from femoral arteries using an RNeasy Mini Kit (Qiagen). RNA 0.5 μg was used to synthesize cDNA using an iScript cDNA Synthesis kit (Bio‐Rad). qPCR reaction was performed using PowerUp^™^ SYBR^®^ Green Master Mix (Thermo Fisher Scientific). The amplifications were performed using an AB 7500 Fast Real‐Time PCR System (Applied Biosystems). The melt curve was also performed and analyzed to avoid any contaminations. BMP2 (Forward: 5′‐TGTGAGGATTAGCAGGTCTTTG‐3′; Reverse: 5′‐TTGTGGAGTGGATGTCCTTTAC‐3′), RUNX2 (Forward: GCCACTTACCACAGAGCTATTA; Reverse: GGCGGTCAGAGAACAAACTA), TAGLN (Forward: AGAGGACTGTAATGGCTTTGG; Reverse: CTGTCTGTGAACTCCCTCTTATG), and GAPDH (Forward: 5′‐GATGCTGGTGCTGAGTATGT‐3′; Reverse: 5′‐GCGGAGATGATGACCCTTT‐3′). The relative mRNA levels were normalized with glyceraldehyde 3‐phosphate dehydrogenase (GAPDH).

### 2.7 Histology and immunohistochemistry

Gastrocnemius muscle and femoral arteries from VitD_3_ or vehicle‐treated rats were fixed with 10% neutral buffered formalin (10% NBF). The tissues were embedded in paraffin, sectioned, and deparaffinized. Von kossa staining was used to assess calcification. H&E staining was used to evaluate the muscle fiber morphology. Verhoeff**–**van Gieson (VVG) staining was used to examine the integrity of elastic fibers in arteries.

To determine capillary density, CD31 immunohistochemistry staining was performed. After deparaffinized, gastrocnemius muscle sections were treated with 10 mM HIER citrate buffer (pH 6.0) for antigen retrieval and then blocked with Dako serum‐free blocking solution (Dako) followed by incubation of anti‐CD31 antibody (Santa Cruz, sc‐1506) and biotinylated anti‐goat secondary antibody. Avidin‐biotinylated enzyme complex (ABC) (Vector Laboratories) and a diaminobenzidine (DAB) substrate chromogen system (Dako) were used to detect CD31. The sections were counterstained with hematoxylin. Slides were viewed with an Olympus microscope with a digital camera. Capillaries and muscle fibers were counted manually by a blinded observer using ImageJ software in 10 different randomized fields per slide (40×) and shown as densities per high power field.

### 2.8 Assessments of arterial stiffness and cardiac function

Pressure–volume parameters were measured under isoflurane (2%) inhalant anesthesia using a 1.4 Fr. micro‐tip pressure–volume catheter (Scisense Inc) inserted into the right common carotid artery (Bae et al., 2016; Lee et al., 2015). Initially, arterial pressure was measured. Measurements of +d*P*/d*T* and −d*P*/d*T* of the blood pressure were used for indirect measurement of arterial stiffness. Then the catheter was gently advanced into the left ventricle to obtain LV hemodynamic parameters. Data were recorded using a Powerlab system (ADInstruments). Beat‐by‐beat pressure–volume parameters including heart rate, stroke volume, +d*P*/d*T*, −d*P*/d*T*, and cardiac output were measured and analyzed using CardioSoft Pro software (CardioSoft).

### 2.9 Statistical analysis

Statistical analysis was performed using SPSS (version 21.0) and GraphPad Prism 6. Data were analyzed by *t*‐test and Mann–Whitney test. *p* < 0.05 was considered to be statistically significant.

## 3 RESULTS

### 3.1 VitD_3_ injection induces medial calcification in the femoral arteries of rats

**FIGURE 1:**
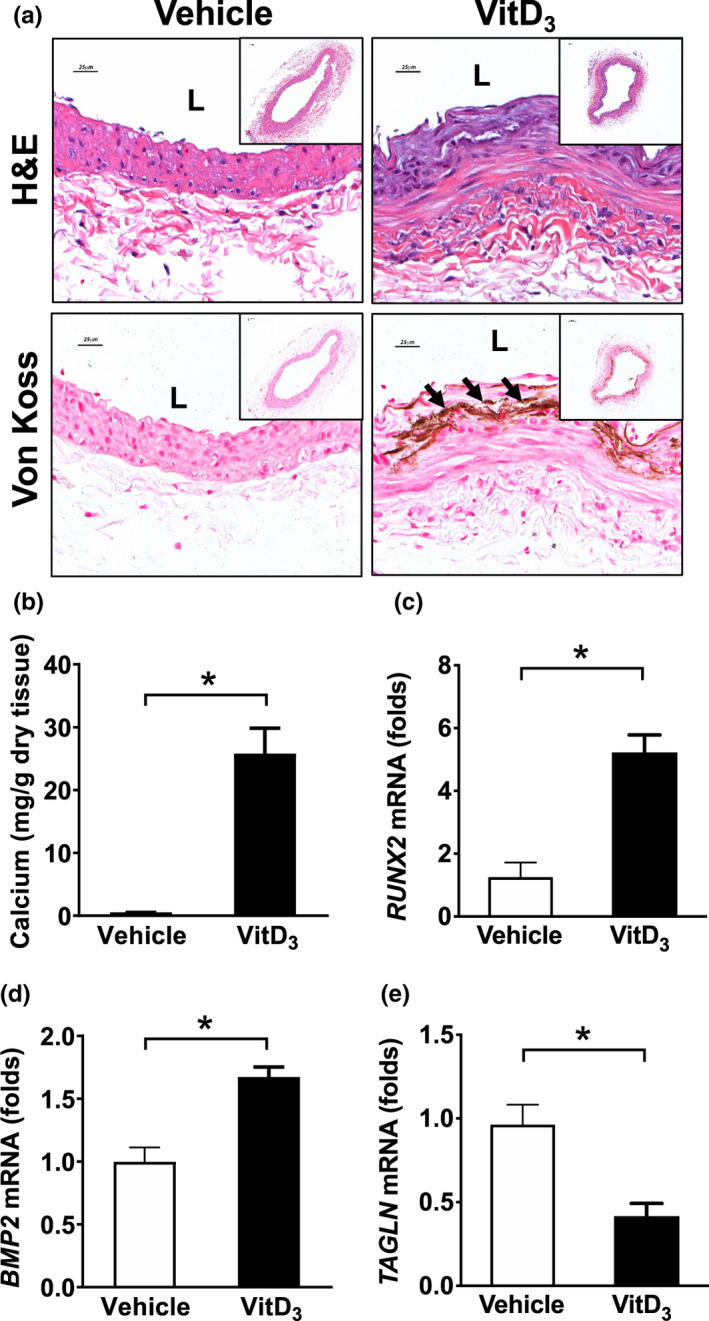
VitD_3_ injection induces medial calcification in the femoral arteries of rats. Male SD rats were SC injected with 3 × 10^5^ IU/kg VitD_3_ or vehicle for 3 consecutive days and the femoral arteries were collected after 14 days. (a) H&E and Von Kossa staining showing that calcification occurred in the medial layers in femoral arteries. (b) Calcium assay showed increased calcium content in VitD_3_‐injected rats compared with vehicle rats. (c) qPCR showed the expression of osteogenic markers *RUNX2* and *BMP2* and SMC marker *TAGLN* in VitD_3_‐injected rats compared with vehicle animals. Values are mean ± SD (*n *= 3). *p < 0.05. L, lumen. Arrow, calcified areas. Scale bar, 25 μm

Administration of VitD_3_ is commonly used to induce medial calcification in rodent models. To confirm that medial calcification was present in lower extremity arteries prior to hindlimb ischemia surgery, rats were subcutaneously injected with vehicle or 3 × 10^5^ IU/kg VitD_3_ as previously described (Qin et al., 2006). As shown in Figure 1a, at 14 days after VitD_3_ injection, vascular calcification was present in superficial femoral arteries but not in those of vehicle‐injected controls. H&E and Von Kossa staining showed that calcification was located in the medial layer. Calcium assay showed increased calcium content in arteries from VitD_3_‐injected animals (Figure 1b). Additionally, qPCR demonstrated increased expression of the osteogenic markers *RUNX2* and *BMP2* and decreased SMC contractile marker *TAGLN* in VitD_3_‐injected rats compared with vehicle‐injected controls (Figure 1c–e). These results indicate that medial calcification develops in lower extremity arteries of rats following VitD_3_ injection.

### 3.2 Medial calcification impairs perfusion after hindlimb ischemia

**FIGURE 2:**
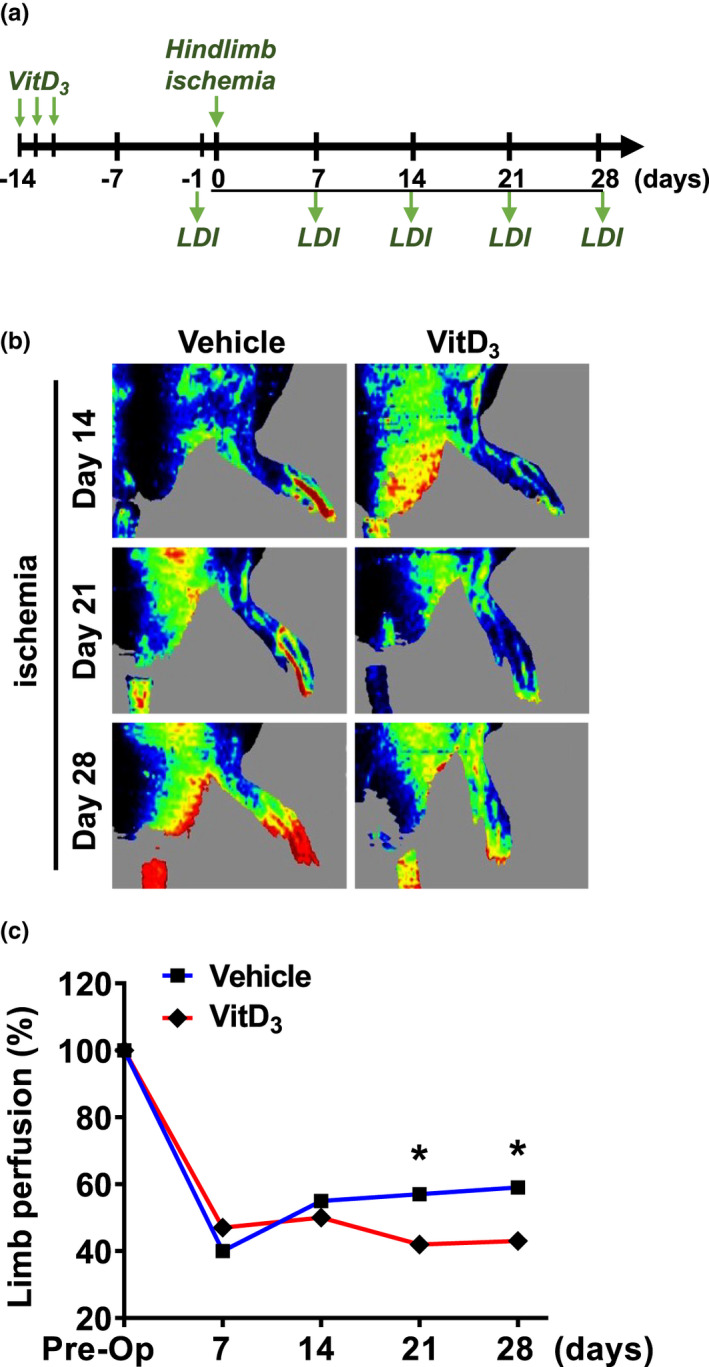
Effects of VitD_3_ injection on limb perfusion after femoral artery ligation. Male SD rats were SC injected with 3 × 10^5^ IU/kg VitD_3_ or vehicle for 3 consecutive days. After 14 days, animals were subjected to femoral ligation. Perfusion of limb tissue was assessed at various time points using Laser Doppler imaging (LDI). (a) Experimental design. (b) Representative images showed the effects of VitD_3_ and vehicle injection on the perfusion following limb ischemia. (c) Quantitative results of Laser Doppler imaging. Values are mean ± SD (*n* = 7–8). *p < 0.05

To study the effects of medial calcification on perfusion in ischemic limbs, rats were first injected with VitD_3_ to induce calcification and then subjected to femoral artery ligation surgery 2 weeks later. Laser Doppler imaging (LDI) was used to assess perfusion before ligation and after 7, 14, 21, and 28 days. As shown in Figure 2, immediately before femoral artery ligation and excision, limb perfusion was not altered in either vehicle‐ or VitD_3_‐injected rats. After left femoral artery occlusion, perfusion was significantly decreased in vehicle‐injected rats at day 7, however, this partially recovered at days 14, 21, and 28. In contrast, there was a minimal recovery of perfusion deficits in VitD_3_‐injected rats.

### 3.3 Medial calcification delays functional recovery following hindlimb ischemia

**FIGURE 3:**
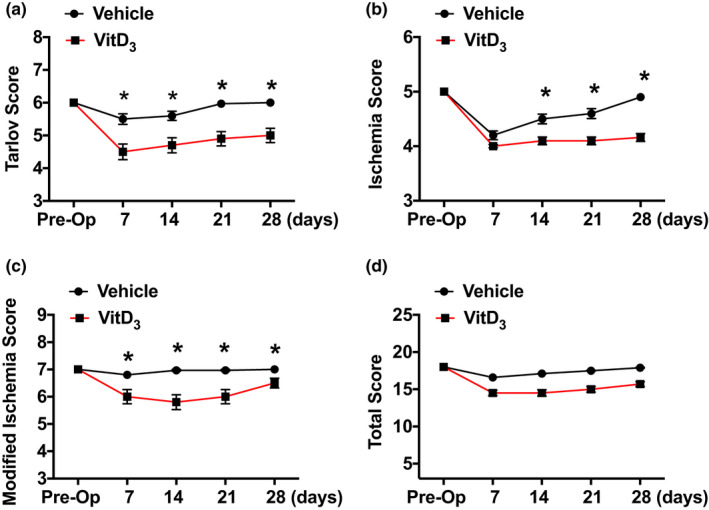
Effects of VitD_3_ injection on functional recovery after femoral artery ligation. Male SD rats were SC injected with 3 × 10^5^ IU/kg VitD_3_ or vehicle for 3 consecutive days. After 14 days, animals were subjected to femoral ligation. Tarlov Score, Ischemia Score, Modified Ischemia Score, or Total Ischemia Score were used to evaluate the function of limbs at various time points. A, Tarlov Score. B, Ischemia Score. C, Modified Ischemia Score. D, Total Ischemia Score. Values are mean ± SEM (*n* = 5). *p < 0.05

Next, we examined the effects of medial calcification on functional recovery after acute limb ischemia. Again, rats injected with vehicle or VitD_3_ underwent femoral artery ligation after 14 days to induce limb ischemia. Tarlov Score, Ischemia Score, Modified Ischemia Score, and Total Ischemia Score were used to evaluate limb function as described previously (Brenes et al., 2012). As shown in Figure 3, after femoral artery ligation, Tarlov Score, Ischemia Score, and Total Ischemia Score were initially decreased in vehicle‐injected rats then gradually returned to baseline. Animals that underwent VitD_3_ injection, however, showed a significantly reduced ability to demonstrate functional recovery.

### 3.4 VitD_3_ injection does not affect capillary and muscle fiber density after femoral artery ligation

**FIGURE 4:**
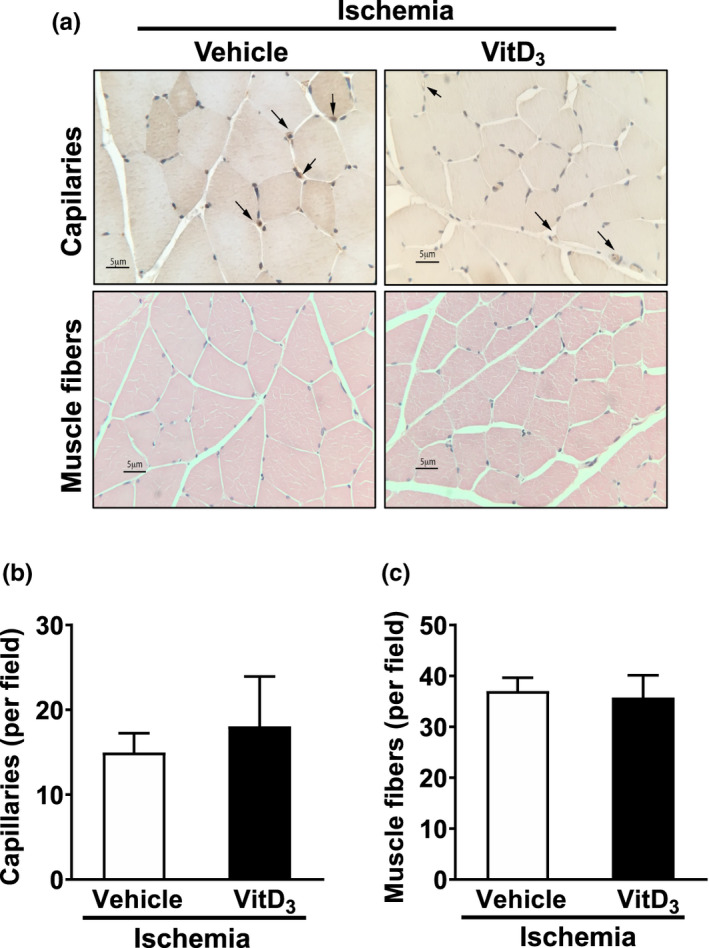
Effects of VitD_3_ injection on capillary density and skeletal muscle morphology after limb ischemia. Male SD rats were SC injected with 3 × 10^5^ IU/kg VitD_3_ or vehicle for 3 consecutive days. After 14 days, animals were subjected to femoral ligation and excision. The gastrocnemius muscles were collected. Capillary density was determined by CD31 immunohistochemical staining, and the morphology of skeletal muscle was examined by H&E staining. (a) Representative images showing effects of VitD_3_ injection on capillary density and morphology of skeletal muscle fibers. (b) Quantitative results of capillary density. Capillary density was defined as number of capillaries per muscle fiber. (c) Quantitative data of skeletal muscle morphology. Values are mean ± SEM (*n* = 6). *p < 0.05

It has been demonstrated that angiogenesis can partially restore limb perfusion after femoral artery ligation in rodent models of acute limb ischemia (Brenes et al., 2012; Iglarz et al., 2001). Limb ischemia‐related angiogenesis is typically assessed in the gastrocnemius muscle. Therefore, we examined the effects of VitD_3_ injection on capillary formation in skeletal muscle fibers following limb ischemia. Capillary density was determined by CD31 immunohistochemical staining. As shown in Figure 4, there were no differences in capillary density between VitD_3_‐ and vehicle‐injected animals at 28 days after femoral artery ligation (Figure 4a, b). Furthermore, no differences in muscle fiber density were detected in VitD_3_‐ versus vehicle‐injected rats after hindlimb ischemia (Figure 4a, c). These findings suggest that decreased perfusion recovery in VitD_3_‐injected rats may not be related to altered angiogenic responses after femoral artery ligation.

### 3.5 VitD_3_ injection increases arterial stiffness and induces elastin degradation in rats

**FIGURE 5:**
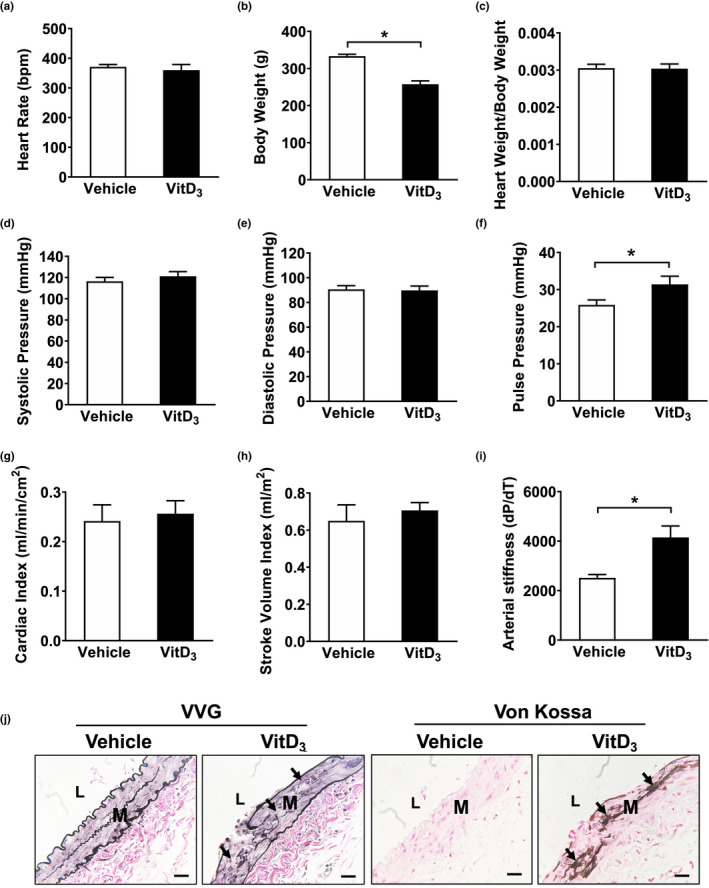
Effects of VitD_3_ on cardiac function, vessel wall compliance, and elastin fiber integrity. Male SD rats were SC injected with 3 × 10^5^ IU/kg VitD_3_ or vehicle for 3 consecutive days. After 14 days, a series of measurements were performed. (a) Heart rate. (b) Body weight. (c) Heart weight/body weight. (d) Systolic pressure. (e) Diastolic pressure. (f) Pulse pressure. (g), Cardiac index. (h) Stroke volume index. (i) Arterial stiffness. (j) The integrity of elastin fibers in femoral arteries was examined by VVG staining, and calcification was determined by Von Kossa staining. Values are mean ± SEM (*n *= 6). *p < 0.05. Arrows indicate the degradation of elastin fibers or medial calcification

We next assessed whether VitD_3_ would affect hemodynamic function in our model. Rats injected with VitD_3_ did not show significant changes in heart rate, heart weight, systolic or diastolic blood pressure, cardiac index, or stroke volume index (Figure 5a, c, d, e, g, h). However, there were significant effects of VitD_3_ on arterial stiffness as measured by central hemodynamics and pulse pressure (Figure 5f, i). To further understand the effects of VitD_3_ on arterial wall characteristics, we examined the integrity of elastin fibers using VVG staining. As shown in Figure 5j, VitD_3_ injection caused medial elastin degradation and disruption that were often collocated with areas where calcification occurred.

## 4 DISCUSSION

The association between medial calcification and worse outcomes in patients with PAD remains unexplained. We here‐in provide data showing that recovery of limb perfusion and function are impaired after femoral artery ligation in a rodent model of medial calcification induced by VitD_3_. This delayed recovery is associated with increased arterial stiffness but not with changes in capillary density, muscle fiber histology, or cardiac function. Although still poorly understood, our findings suggest that medial artery calcification as seen in patients with diabetes and renal disease may lead to worse outcomes at least in part, through its association with decreased arterial compliance. Strategies aimed at reducing or slowing the progression of medial calcification may thus improve limb outcomes in patients who develop PAD.

Prior investigations have demonstrated an association between medial calcification and poor outcomes in patients with PAD (Ho & Shanahan, 2016). Recent clinical studies have shown an association between arterial calcification and amputation in patients with chronic limb‐threatening ischemia (Chantelau et al., 1995; Guzman et al., 2008). In a large study of more than 1000 Finnish patients with non‐insulin‐dependent diabetes, medial calcification was found to be a strong predictor of cardiovascular events and amputation (Lehto et al., 1996b). In the present study, rats were first injected with VitD_3_ to induce medial calcification, and then subjected to hindlimb ischemia by ligation and excision of a segment of femoral artery. We found that recovery from hindlimb ischemia, in terms of both perfusion and function, was significantly worse in rats injected with VitD_3_. In this model, perfusion and limb function are decreased immediately after femoral artery ligation with gradual recovery that depends on the extent of vascular interruption. Our findings in vehicle‐injected rats are similar to those of Brenes et al. (2012) who observed an immediate decrease in both functional recovery scores and perfusion measures after femoral artery ligation in mice, with gradual recovery to 28 days. In our studies, rats injected with VitD_3_ to induce medial calcification showed an inability to recover limb perfusion as measured by laser Doppler. We also noted a decreased ability to recover limb function as assessed by Tarlov parameters.

We next considered multiple factors that could be responsible for these effects. It has been shown in rodent models of hindlimb ischemia, as well as in patients with PAD, that angiogenesis as evidenced by increased capillary formation can occur in ischemic tissues. This is triggered by reduced oxygen delivery and thought to favor improved perfusion and functional recovery. Inhibition of capillary formation can slow the recovery process (Brenes et al., 2012; Luo et al., 2002; Niiyama et al., 2009). In human studies, decreased perfusion of the soleus and gastrocnemius muscle inversely correlates with the severity of symptoms in patients with symptomatic PAD (Lindner et al., 2008). In the present study, however, we were unable to demonstrate a difference in capillary density between VitD_3_‐ and vehicle‐injected rats at 28 days after femoral artery ligation, 6 weeks after VitD_3_ injections. It remains possible that this inability to show a difference was related to missing the relevant time points during earlier phases of ischemia, insufficiently sensitive methods, or the high statistical variability of our model. We were also unable to identify any differences in muscle fiber density between the two groups at 28 days after vessel ligation and again note that this may be related to the late time point of these analyses compared with other investigations. Further efforts to assess the effects of VitD_3_, medial calcification, and femoral artery ligation on angiogenic and muscle fiber responses are thus warranted.

We next asked whether the delayed recovery observed in VitD_3_‐injected rats could be related to cardiotoxic effects of VitD_3_. Although deficiency of VitD_3_ is the most common clinical problem, cardiotoxic effects of VitD_3_ overdose have been demonstrated in rodent models (Lugg et al., 2015; Melamed & Thadhani, 2012; Muscogiuri et al., 2017). These include left ventricular hypertrophy, hypertension, shortened QT interval, and ST‐segment elevation (Marcinowska‐Suchowierska et al., 2018). Moreover, rats treated with an excessive dose of VitD_3_ are more likely to develop aortic atherosclerosis (Kunitomo et al., 1981). Additionally, administration of high dose of VitD_3_ (3000 IU/kg) is associated with significant renal injury in obese mice, although it provides metabolic benefits including elevated insulin sensitivity and decreased body weight (Gaspar et al., 2017). In the present study, we used 3 × 10^5^ IU/kg of VitD_3_ to induce medial calcification in rats. We were unable to demonstrate significant changes in heart rate, systolic or diastolic pressure, cardiac index, or left ventricular wall thickness in VitD_3_ versus control rats suggesting that the effects of cholecalciferol on recovery of perfusion were unrelated to cardiac function.

We then assessed the effects of VitD_3_ on arterial compliance measures. Previously, it has been shown that increased stiffness and resistance measures are associated with decreased popliteal artery blood flow in patients with diabetes (Suzuki et al., 2009). Niederhoffer et al. (1997), showed a relationship between elastin degradation and increased arterial stiffness, and suggested that medial calcification‐induced destruction of elastin fibers that led to arterial stiffness along with left ventricular hypertrophy. Furthermore, Sutliff et al evaluated the effect of medial calcification on vascular function in vivo and demonstrated decreased compliance (Sutliff et al., 2011). These findings are consistent with the increased pulse wave velocity and elastin degradation seen in uremic animal models of medial calcification (Niederhoffer et al., 1997; Noonan et al., 2008). Maizel et al. (2009) observed increased arterial stiffness together with left ventricle hypertrophy in the uremic mice and suggested that medial rather than intimal calcification reduced arterial compliance. Our data showed a significant increase in arterial stiffness associated with destruction of elastin fibers in VitD_3_‐injected rats. This was noted both by measurement of changes in +d*P*/d*T* and −d*P*/d*T* of blood pressure (Figure 5f, i), as well as destruction of elastic fibers on VVG staining (Figure 5e). We also observed that medial calcification primarily occurred in the area of elastic fiber degradation after VitD_3_ injection (Figure 5e, arrow). Combined with findings from others, these data suggest that elastin fiber degradation may be one of the mechanisms by which VitD_3_‐induced calcification could increase arterial stiffness.

It remains unclear how such vascular changes could affect limb perfusion. Aortic stiffening has been associated with increased peripheral vascular resistance, and such changes can lead to decreased limb perfusion (Guo & Kassab, 2020). Moreover, human studies have demonstrated a decreased vasodilatory response in patients with significant coronary calcification (Wang et al., 2006). A more recent study has demonstrated that increased arterial stiffness is an important predictor of mortality in patients with claudication or chronic limb‐threatening ischemia (Mendes‐Pinto & Rodrigues‐Machado, 2019). Taken together, these data suggest that arterial stiffness caused by medial calcification may play a role in the observed reduction in blood flow, and function either via a direct effect on perfusion or through indirect effects on distal vessel resistance. It is worth noting that we employed a surgical model of hindlimb ischemia to mimic PAD in this study. Although this is a commonly used animal model, its clinical relevance remains uncertain (Zhuang et al., 2011).

In summary, using a rat model of acute limb ischemia, we found that injection of VitD_3_ to induce medial calcification was associated with impaired recovery of limb perfusion and function. While the underlying mechanisms are complex, it appears that increased arterial stiffness may play a role. This study provides impetus into efforts focused on inhibiting medial calcification to improve outcomes in patients with PAD.

## CONFLICTS OF INTEREST

None.

## AUTHOR CONTRIBUTIONS

SZ, YC, and RG designed the experiments; SZ, XW, SM, TL, and YC performed the experiments. SZ, XW, SM, and YC analyzed the data; SZ, YC, and RG wrote and revised the manuscript.
